# Intelligence Classification Algorithm-Based Drug-Resistant Pulmonary Tuberculosis Computed Tomography Imaging Features and Influencing Factors

**DOI:** 10.1155/2022/3141807

**Published:** 2022-05-19

**Authors:** Yanping Jiang, Xinguo Zhao, Zhengfei Fan

**Affiliations:** ^1^Department of Tuberculosis, Wuxi No.5 People's Hospital, Wuxi 214000, Jiangsu, China; ^2^Department of Emergency, Huzhou Central Hospital, Huzhou 313000, Zhejiang, China; ^3^Department of Clinical Medicine, Wenzhou Medical University, Wenzhou 325000, Zhejiang, China

## Abstract

The drug resistance and influencing factors of patients with pulmonary tuberculosis were investigated, and a dual attention dilated residual network (DADRN) algorithm was proposed. The algorithm was applied to process and analyze lung computed tomography (CT) images of 400 included patients with pulmonary tuberculosis. Besides, sparse code book algorithm and bag of visual word (BOVW) algorithms were introduced and compared, and the influencing factors of pulmonary tuberculosis drug resistance were analyzed. The results demonstrated that the localization precision of lung consolidation, nodules, and cavities by the DADRN algorithm reached 91.2%, 92.5%, and 93.8%, respectively. The recall rate of the three algorithms amounted to 83.55%, 84.5%, and 86.4%, respectively. Both localization precision and recall rate of the DADRN algorithm were higher than those of other two algorithms (*P* < 0.05). The drug resistance rate of streptomycin, isoniazid, and rifampin of the patients aged between 40 and 59 was all higher than those of the patients in other age groups. The drug resistance rate of streptomycin, isoniazid, and rifampin of retreated patients was all higher than those of patients initially treated. The drug resistance rate of streptomycin, isoniazid, and rifampin of the patients with tuberculosis contact was all higher than those of the patients without tuberculosis contact (*P* < 0.05). Based on the above results, the accuracy of CT images processed by dual attention-based dilated residual classification network algorithm was higher than that processed by other two algorithms. Age, medical history, and history of exposure to tuberculosis were the influencing factors of the drug resistance of patients with pulmonary tuberculosis.

## 1. Introduction

Tuberculosis is an ancient disease with a history of more than 4000 years, and it still seriously threatens human health [1]. With the infection of the human immunodeficiency virus, the increase in the floating population, and the decline of the body's immune level, the drug resistance of Mycobacterium tuberculosis continues to increase, and tuberculosis spreads around the world. People begin to pay attention to the drug-resistance phenomenon of Mycobacterium tuberculosis [2, 3]. Pulmonary tuberculosis is a dangerous and incurable disease with a long course. It increases mortality among patients and causes negative impacts on patients' life and safety. Mycobacterium tuberculosis is the main cause of chronic infectious lung diseases [4]. The drug-resistance rate of pulmonary tuberculosis continues to increase, and drug-resistance strains continue to spread, making treatment more difficult. It is very important to formulate a reasonable and effective diagnosis and treatment plan [5].

There are many lesions in pulmonary tuberculosis, among which the basic ones include exudative, proliferative, caseous, pulmonary cavities, fibrosis, and calcification. However, many lesions are not typical on imaging images, and it is difficult to identify [6]. The diagnostic methods for pulmonary tuberculosis patients mainly include chest X-ray examination, computed tomography (CT) examination, and magnetic resonance imaging (MRI). At present, chest X-ray is the common one [7]. However, there are tissues with high overlapping density around the lung tissue, and the resolution is low. It is difficult to clearly display the lesion tissue and the shape of the lesion by X-ray, which has certain limitations [8]. Some studies reveal that CT can detect misdiagnosed cases by X-ray and determine if pulmonary tuberculosis is active, which shows a very high application value of CT. Hence, it is widely applied in clinical practice [9]. In recent years, the application of artificial intelligence technology to computer-aided diagnosis has become a hot topic of exploration, and intelligent classification of lung lesions is an important issue [10]. The existing automatic classification algorithms for pulmonary lesions must first label the lesion area in the image and then classify the extracted features of the lesion area. The workload of image labeling is large, and the efficiency is low [11]. Compared with other images, medical images have the characteristics of small sample size and difficulty in labeling, which reduces the workload of labeling and improves the efficiency of classification, which has become a current research hotspot [12].

A dual attention-based dilated residual classification network algorithm was proposed. The algorithm was aimed at establishing a global dependency relationship for a high-level semantic feature map from two dimensions, including the position attention module and channel attention module, which enables similar positions or channels to gain each other. The algorithm could automatically enhance the feature differences of normal lung tissue and tuberculosis lesion tissue and predict as well as judge the types of lesions. A set of fully automatic high-precision solution provided the reference for the prevention of the drug resistance of pulmonary tuberculosis patients.

## 2. Materials and Methods

### 2.1. Research Objects

A total of 400 pulmonary tuberculosis patients admitted to the hospital between January 2018 and June 2021 were selected, including 206 males and 194 females aged between 19 and 75. The patients included were treated with pulmonary tuberculosis drugs, including streptomycin, rifampicin, and isoniazid, and the drug resistance of different drugs in the patients was counted. All the patients and their families understood the study and signed informed consent. This study had been approved by the ethics committee of the hospital.

Inclusion criteria were as follows: patients with a complete medical history and imaging data; patients without mental disorder; patients without genetic disease; patients and their family members who had signed informed consent forms.

Exclusion criteria were as follows: patients who combined with major organ diseases; patients who combined with malignant tumors; patients who are unwilling to participate in the research.

### 2.2. CT Examination

A 64-slice spiral CT scanner was used to scan patients' chests. The scanning method was double-slice spiral CT. Scanning parameters were set as follows. Tube voltage was 120 kV, tube current was 45 mAs, layer thickness amounted to 5.0 mm, and pitch was 1.25 mm.

### 2.3. CT Image Lesion Localization Based on DADRN Algorithm

#### 2.3.1. Image Preprocessing

Usually, one scan of each patient's lung CT will generate dozens or even hundreds of files, each corresponding to a two-dimensional slice image. The views of the CT image in three standard directions were set as (*xy*, *yz*, *xz*), and the related data of the Dicom file can be directly read through pydicom plug-in, including the original gray value, slope value, and intercept value. The CT value corresponding to the Dicom image *a* was set to *H*_*a*_, and the calculation method was shown in equation (1). In the equation, *G*_*a*_ represented the original gray value corresponding to Dicom image *a*, *S*_*a*_ represented the slope corresponding to Dicom image *a*, and *T*_*a*_ referred to the intercept corresponding to Dicom image *a*.

(1) $$\begin{array}{c} H_{a}=G_{a}*S_{a}+T_{a}. \end{array}$$

The CT value of the CT image was truncated according to the window width and window level in the file. WCa represented the window level corresponding to Dicom image *a*, *Ww*_*a*_ represented the window width corresponding to Dicom image *a*, *L*_*a*_ referred to the lower bound of the truncation range, and *U*_*a*_ was the upper bound of the truncation range. The calculation method was shown in equations (2) and (3), and the cut-off range was expressed as [*L*_*a*_, *U*_*a*_].

(2) $$\begin{array}{c} L_{a}=WC_{a}-\frac{1}{2}WW_{a}, \end{array}$$

(3) $$\begin{array}{c} U_{a}=WC_{a}+\frac{1}{2}WW_{a}. \end{array}$$

The fixed resolution used was 1 mm × 1 mm × 1 mm. *I*_*ab*_ represented the pixel value at coordinates [*a*, *b*] in the image, and the calculation method was shown in the following equation:

(4) $$\begin{array}{c} I_{ab}=\frac{I_{ab}-L_{a}}{U_{a}-L_{a}}. \end{array}$$

#### 2.3.2. Image Segmentation

**Figure 1:**
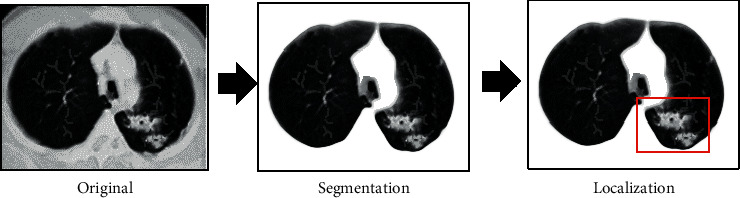
Flow chart of lung CT image segmentation and lesion localization.

The simple threshold method is difficult to automatically segment the lungs from the CT images. In this work, the postprocessing optimization was carried out through the fully connected conditional random field, and the segmentation results of the liver organs were further improved. The flowchart of image segmentation and lesion localization was shown in Figure 1.

#### 2.3.3. Dual Attention Mechanism

The positional attention module establishes long-distance dependencies for any two points in the spatial position, captures global similar features, and utilizes global information when synthesizing output features. The input high-dimensional semantic feature map was set to be *M*∈*R*^*C*× *H* × *W*^, and the input feature *M* was mapped into two feature spaces *E*_1_, *E*_2_∈ℝ^*C*1×(*H* × *W*)^ through 1 × 1 convolutional layer and batch normalization layer. It could multiply the transpose of the matrix *E*_1_ and the matrix *E*_2_ and normalize it with the sigmoid function to obtain the global position similarity matrix *α*_*ba*_∈ ℝ^*N* × *N*^. Among them, *N* = *H* × *W*, *α*_*ab*_ represented the similarity between the position *a*^th^ and the position *b*^th^, and the calculation method was shown in equation (5). Similarity represented the cosine distance of any two locations in space. In fact, the result of matrix multiplication was equal to the cosine distance of each position and all positions in space. There were eigenvectors *M* and eigenvectors *E*, and the calculation method of cosine distance was shown in equation (6).

(5) $$\begin{array}{c} \alpha_{ba}=\frac{1}{1+e^{-S_{ab}}},S_{ab}=E_{1}^{T}E_{2}, \end{array}$$

(6) $$\begin{array}{c} \text{cosine}\_\text{sim}\left(M,E\right)=\frac{M\cdot E}{ME}. \end{array}$$

After the input feature *M* was passed through a 1 × 1 convolutional layer, a feature map *O*∈ℝ^*C* × *H* × *W*^ was obtained, and the dimension of the feature map remained unchanged. Multiplying the normalized global position similarity matrix *α* and the feature map *O* was the new position attention feature map *P*_*b*_, and the calculation method was shown in equation (7). When the feature vector of the position *b*^th^ of *P*_*b*_ was synthesized, the feature information of all other positions would be integrated, and *α*_*a,b*_ represented the importance of the position *a*^th^ when the feature vector of the position *b*^th^ was synthesized.

(7) $$\begin{array}{c} P_{b}=\sum_{a=1}^{N}\alpha_{b,a}O_{a}. \end{array}$$

Finally, the output of the position attention module was *y*∈ℝ^*C* × *H* × *W*^, which represented the result of the element-level addition of the original feature map and the new synthetic feature map. The calculation method was shown in equation (8). *β* was an adaptive parameter, which was initialized to 0 at the beginning of training, and its weight was continuously increased through network backpropagation.

(8) $$\begin{array}{c} y_{b}=\beta P_{b}+M_{b}. \end{array}$$

The channel attention module emphasizes the dependencies between learning channels. The two-dimensional feature map corresponding to each channel is regarded as the response corresponding to a category. The channel attention module is computed directly based on the high-level semantic features *M*. First, the input feature *M* was directly changed from ℝ^*C* × *H* × *W*^ to ℝ^*C* × (*H* × *W*)^, then the transformed *M* and its transpose were multiplied, and the sigmoid activation function was adopted to normalize the result to get outgoing channel global similarity matrix. The calculation method was given in the following equation:

(9) $$\begin{array}{c} y_{ba}=\frac{1}{1+e^{-\left(M_{a}^{\prime }M_{b}\right)}},y\in R^{c\times c}. \end{array}$$

**Figure 2:**
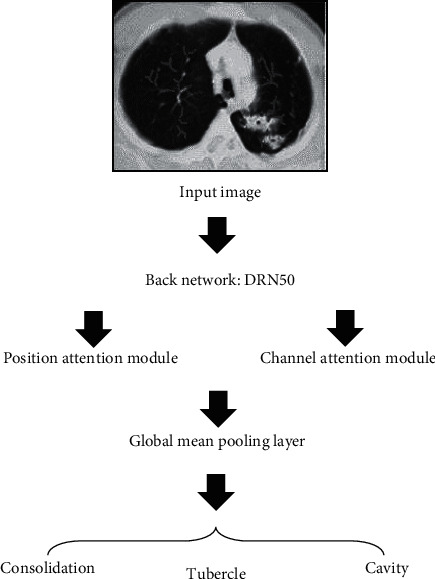
Flowchart based on DADRN algorithm.

Then, the feature *M* and the channel global similarity matrix *y* were multiplied to obtain a new synthetic feature map. The synthetic result was multiplied by the adaptive learning parameter *μ*, the result and the original input feature map *M* were performed with element-wise addition, and the output of the final channel attention module was *x*∈ℝ^*C* × *H* × *W*^ (equation (10)). The output of the location attention and channel attention modules was superimposed in the channel dimension to obtain the feature *z*∈ℝ^(*C* × 2) × *H* × *W*^, which was input into the classification branch to obtain the final classification result. The specific flow chart of the algorithm was shown in Figure 2.

(10) $$\begin{array}{c} x_{b}=\mu \overset{c}{\underset{a=1}{\sum }}\left(y_{ba}M_{a}\right)+M_{b}. \end{array}$$

#### 2.3.4. Objective Function

The weighted cross-entropy function was selected as the objective function of the network. This function can assign different weight values to samples of different categories when the loss function was calculated according to the number of samples of different categories. The calculation method of the normal binary cross-entropy loss function was as equation (11). In order to make the model close to the distribution of the real data, the minimized KL divergence between the model data distribution and the training data was undertaken as a supervised loss function, where *r* represented the true label of the data, and *p* represented the model output prediction result. The weighted cross-entropy function would weight a class with a smaller number of samples, as shown in equation (12). The positive and negative samples showed different weights. The weights of positive and negative samples were normalized, as shown in equation (13), where *A* represented the number of negative samples, and *B* referred to the number of positive samples.

(11) $$\begin{array}{c} L_{\text{CE}}=-r\;\log\;p-\left(1-r\right)\log\left(1-p\right), \end{array}$$

(12) $$\begin{array}{c} L_{\text{WCE}}=-W_{0}r\;\log\;p-W_{1}\left(1-r\right)\log\left(1-p\right), \end{array}$$

(13) $$\begin{array}{c} w_{0}=\frac{A}{A+B},w_{1}=\frac{B}{A+B}. \end{array}$$

### 2.4. Observation Indicators

The lung lesion classification accuracy, precision, recall rate, and *F*1-score of the three algorithms were calculated, respectively. The main categories were consolidation, nodules, and cavities. It was assumed that the class of interest was the positive class, and the other classes were the negative class. According to the correctness of the prediction result of the classifier, it was divided into four cases: TP meant predicting positive samples as positive, FN meant predicting positive samples as negative, FP meant predicting negative samples as positive, and TN meant predicting negative samples as negative. The calculation methods of the four evaluation indicators were shown in equations (14)–(17), respectively, where *β* = 1 was the *F*1-score.

(14) $$\begin{array}{c} \text{Accuracy}=\frac{\text{TP}+\text{TN}}{\text{TP}+\text{FN}+\text{FP}+\text{TN}}, \end{array}$$

(15) $$\begin{array}{c} \text{Precision}=\frac{\text{TP}}{\text{TP}+\text{FP}}, \end{array}$$

(16) $$\begin{array}{c} \text{Recall}=\frac{\text{TP}}{\text{TP}+\text{FN}}, \end{array}$$

(17) $$\begin{array}{c} F\beta =\left(1+\beta^{2}\right)\frac{\left(1+\beta^{2}\right)\text{TP}}{\left(1+\beta^{2}\right)\text{TP}+\beta^{2}\text{FP}+\text{FN}}. \end{array}$$

The drug resistance of the patients included was counted, and the selected drugs were mainly streptomycin, isoniazid, and rifampicin. The drug-resistance influencing factors of pulmonary tuberculosis patients were analyzed, including gender, age, medical history, and history of exposure to tuberculosis.

### 2.5. Statistical Analysis

SPSS 20.0 software was used for the statistical analysis of data, and a *t*-test was used. The count data were expressed as a rate (%), and *P* < 0.05 meant the difference was statistically significant.

## 3. Results

### 3.1. Comparison of Accuracy and Precision of Lesion Diagnosis in Different Types of Pulmonary Tuberculosis Patients by Three Algorithms

**Figure 3:**
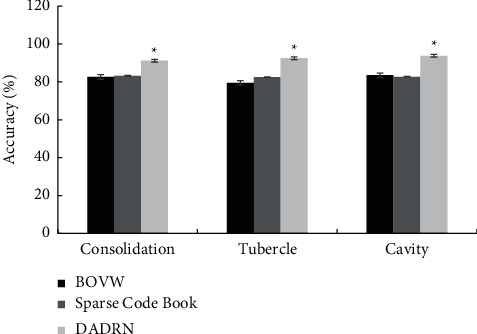
Comparison of lesion localization correct rate of different types of pulmonary tuberculosis patients by three algorithms. ^*∗*^ represents the comparison with the other two algorithms, *P* < 0.05.

**Figure 4:**
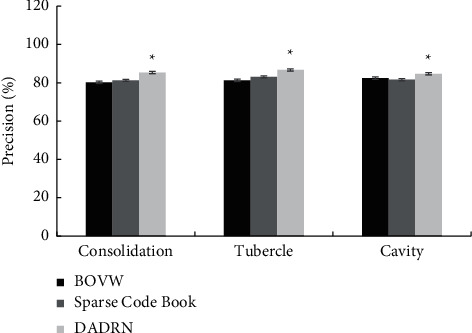
Comparison of lesion localization precision of different types of pulmonary tuberculosis patients by three algorithms. ^*∗*^ indicates the comparison with the other two algorithms, *P* < 0.05.

Figure 3 shows the comparison of the accuracy of lesion localization for different types of pulmonary tuberculosis patients using the three algorithms.  Figure 4 illustrates the comparison of the localization precision of different types of pulmonary tuberculosis patients by the three algorithms. Compared with the SCB algorithm and the BOVW algorithm, the DADRN algorithm showed higher positioning accuracy and precision for lung consolidation, nodules, and cavities, and the differences were statistically significant (*P* < 0.05).

### 3.2. Comparison of Diagnostic Recall Rate and F1-Score of Lesions in Different Types of Pulmonary Tuberculosis Patients by Three Algorithms

**Figure 5:**
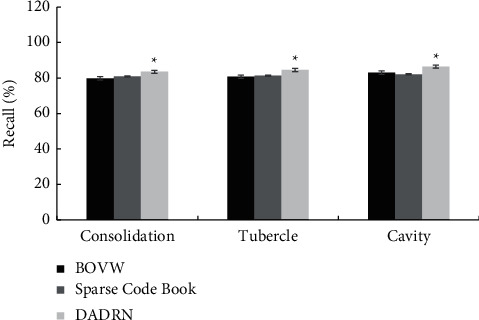
Comparison of lesion localization recall rate of different types of pulmonary tuberculosis patients by three algorithms. ^*∗*^ shows the comparison with the other two algorithms, *P* < 0.05.

**Figure 6:**
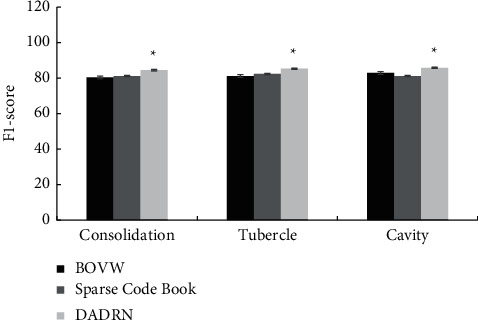
Comparison of lesion localization *F*1-score of different types of pulmonary tuberculosis patients by three algorithms. ^*∗*^ suggests the comparison with the other two algorithms, *P* < 0.05.

Figure 5 shows the comparison of the recall rate of lesion localization in different types of pulmonary tuberculosis patients by the three algorithms.  Figure 6 shows the comparison of the *F*1-score of the lesion location of different types of pulmonary tuberculosis patients by the three algorithms. Compared with the SCB algorithm and the BOVW algorithm, the DADRN algorithm showed a higher diagnostic recall rate and *F*1-score for lung consolidation, nodules, and cavities, and the differences were statistically significant (*P* < 0.05).

### 3.3. Statistics of Drug-Resistance Rate of Three Drugs in Pulmonary Tuberculosis Patients

**Figure 7:**
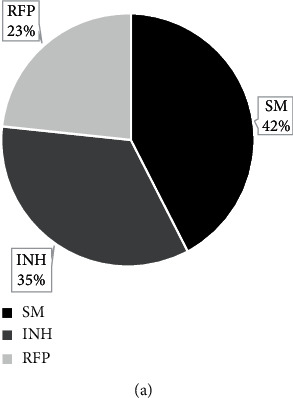
Statistics of the drug-resistance rate of three drugs in pulmonary tuberculosis patients.

Figure 7 shows the statistics of the drug-resistance rate of three drugs in pulmonary tuberculosis patients. Among the 400 pulmonary tuberculosis patients included, there were 52 drug-resistance patients, and the overall drug-resistance rate was 13%. Among them, there were 22 patients with streptomycin resistance, the drug-resistance rate was 5.5%, and the proportion of drug-resistance patients was 42%. There were 18 patients with isoniazid resistance, the drug-resistance rate was 4.5%, and the proportion of drug-resistance patients was 35%. There were 12 patients with rifampicin drug resistance, the drug-resistance rate was 3%, and the proportion of drug-resistance patients was 23%. It can be concluded that the streptomycin resistance rate was higher, and the rifampin resistance rate was the lowest.

### 3.4. Statistics on Influencing Factors of Drug Resistance for Pulmonary Tuberculosis Patients

**Figure 8:**
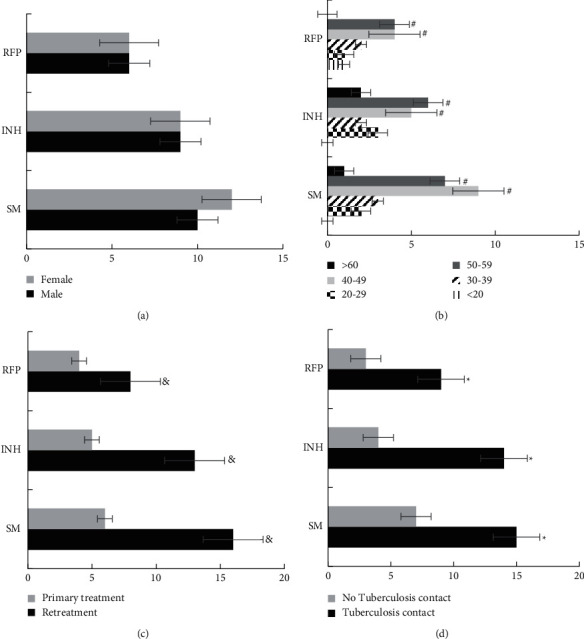
Statistics on influencing factors of the incidence of drug resistance of pulmonary tuberculosis patients. (a) The influence of gender on drug resistance; (b) the influence of age on drug resistance; (c) the influence of medical history on drug resistance; (d) the influence of pulmonary tuberculosis contact on drug resistance. # indicates the comparison with other age groups, *P* < 0.05. ^*∗*^ represents the comparison with the patients receiving primary treatment, *P* < 0.05. & denotes the comparison with the patients without pulmonary tuberculosis contact, *P* < 0.05.

Figure 8 shows the statistics of influencing factors of drug resistance for pulmonary tuberculosis patients. Among them, *A* showed the effect of gender on drug resistance, *B* was the effect of age on drug resistance, *C* was the effect of medical history on drug resistance, and *D* showed the effect of history of exposure to tuberculosis on drug resistance. It can be found that there was no significant difference in the drug resistance of pulmonary tuberculosis patients of different genders, which was not statistically significant (*P* > 0.05). The drug resistance of pulmonary tuberculosis patients aged 40–59 was significantly higher than that of other age groups. The drug resistance of the three drugs in retreated patients was significantly higher than that of patients initially treated, and the drug resistance of the three drugs in patients with a history of exposure to tuberculosis was significantly higher than that in patients without a history of exposure to tuberculosis, showing statistically significant differences (*P* < 0.05).

## 4. Discussion

Pulmonary tuberculosis is a specific infectious disease with a high incidence in young and elderly people [13]. The main clinical manifestations of patients are low-grade fever, cough, night sweats, chest pain, etc. If the treatment is not timely, it will seriously affect their physical and mental health [14]. In recent years, due to the extensive use of antibiotics, the drug resistance of Mycobacterium tuberculosis has been increasing, and the efficacy of drugs has become more and more limited [15]. Patients with pulmonary tuberculosis generally have low immunity. If they are not diagnosed and treated in a timely manner, serious complications such as respiratory tract infection and severe pneumonia will occur, threatening the life safety of patients [16]. The long-term stimulation of pulmonary tuberculosis in patients can cause metaplasia of the lesion tissue and adjacent parts of the epithelial tissue, stimulate cell proliferation and spread, and easily develop into lung cancer. Therefore, early diagnosis and treatment are extremely important [17, 18]. CT shows the advantages of fast scanning speed, clear images, and high security, provides a large amount of information, and is widely used in clinical practice [19].

The lung lesion automatic classification algorithm shows positive significance in clinical adjuvant diagnosis. Facing a large amount of medical imaging data, the support from the computer-aided diagnostic system is very important [20]. With the continuous development of modern medical imaging equipment technology, it becomes a common method to initially diagnose and classify diseases based on digital imaging data [21]. The current classification algorithm relies heavily on the fine labeling of lesion regions, which increases the workload of data labeling and causes inconvenience to operation. Some researchers [22] put forward an integrated deep learning-based adjuvant diagnosis algorithm. Stacked generalization ensemble learning was combined with VGG16 deep learning to form cascade connection classifiers to classify and verify novel coronavirus pneumonia patients, common pneumonia patients, and normal control patients. The predictive accuracy, sensitivity, specificity, and precision of the algorithm were 93.57%, 94.21%, 93.93%, and 89.40%, respectively. Zhang et al. [23] proposed a CT image hybrid feature-based pulmonary nodule classification method. Besides, they fused 3D deep  dual-path network (DPN) features, local binary pattern (LBP)-based texture features, and histogram of directional gradient (HOG)-based shape features to characterize pulmonary nodules. The method shows very significant robustness in the classification of nodule pictures and can help radiologists interpret diagnostic data and make decisions in clinical practice. Xu et al. [24] proposed a texture specificity BoVW method and a spatial cone matching (SCM)-based presentation strategy to represent focal hepatic lesions. The discovery of texture specificity SCM-based BoVW features could effectively represent various hepatic lesions and improve the diagnostic accuracy of radiologists. A dual attention-based dilated residual classification network algorithm was proposed to automatically classify the lesions of CT images of patients with the weakly labeled pulmonary nodule. The dilated residual network fused with a dual attention mechanism could capture global features. It not only could enhance the correct rate and precision of classification but also improve the localization accuracy of weakly labeled lesions.

The classification of lesions in CT images of pulmonary tuberculosis patients was explored based on the DADRN algorithm, and the accuracy, accuracy, recall rate, and *F*1-score of three algorithms for the classification of pulmonary tuberculosis lesions were compared. The drug resistance and influencing factors of pulmonary tuberculosis patients were analyzed. The results showed that the accuracy, precision, recall rate, and *F*1-score of the DADRN algorithm for localization of lung consolidation, nodules, and cavities were higher than those of the SCB algorithm and BOVW algorithm, and the differences were statistically significant (*P* < 0.05). The total drug-resistance rate of pulmonary tuberculosis patients included was 13%, the drug-resistance rate of streptomycin was higher, and the rifampin resistance rate was the lowest. There was no significant difference in the drug resistance of pulmonary tuberculosis patients of different genders (*P* > 0.05). The drug-resistance rate of pulmonary tuberculosis patients aged 40–59 was significantly higher than that of other age groups. The drug resistance of the three drugs in retreated patients was significantly higher than that of patients initially treated, and the drug resistance of the three drugs in patients with a history of exposure to tuberculosis was significantly higher than that in patients without a history of exposure to tuberculosis, showing statistically significant differences (*P* < 0.05). The DADRN algorithm can be applied to the intelligent classification of lung CT image lesions in pulmonary tuberculosis patients and has good application effects.

## 5. Conclusion

A dual attention-based dilated residual classification network algorithm could realize intelligent classification of the lesions of CT images of pulmonary tuberculosis patients with high accuracy. The algorithm could be applied in clinical practice to reduce workload and improve the processing efficiency of CT images. Hence, it could be promoted and applied in clinical diagnosis and treatment. The disadvantage of the research lies in the small sample size. Further studies and verification are necessary. The sample size should be enlarged and compared with other algorithms to seek an optimal intelligent classification method of lung CT image lesions.

## Data Availability

The data used to support the findings of this study are available from the corresponding author upon request.
